# First-Principles Molecular Dynamics Simulations of Infrared and Raman Vibrational Spectra of H_5_O_2_^+^, D_5_O_2_^+^, DH_4_O_2_^+^, and D_4_HO_2_^+^ from 50 to 300 K

**DOI:** 10.1021/acs.jpca.5c05184

**Published:** 2025-10-22

**Authors:** Oluwaseun Omodemi, Martina Kaledin

**Affiliations:** Department of Chemistry & Biochemistry, 15617 Kennesaw State University, 370 Paulding Ave NW, Box # 1203, Kennesaw, Georgia 30144, United States

## Abstract

We report molecular dynamics simulations of infrared (IR) and Raman spectra of H_5_O_2_^+^ and its deuterium-substituted analogs. We use the well-tested HBB potential and dipole surfaces along with a recently fitted polarizability surface of CCSD­(T)/aug-cc-pVTZ quality. The focus of the present work is to provide new insights into the O···O stretch region, located in the [500–700] cm^–1^ spectral range, by means of analyzing the spectra over a broad range of temperatures: from 50 to 300 K. Also, rovibrational thermal averaging was performed to untangle the unusually complex spectra of partially deuterated isotopologues DH_4_O_2_^+^ and D_4_HO_2_^+^ with H and D minority species in both internal (int) and external (ext) positions. Our findings show that DH_4_O_2_^+^ (ext) is at least 90% prevalent at the [50–300] Kelvin temperatures, predominantly due to its low zero-point energy (ZPE). Furthermore, contrary to previous reports that the mixed isotopologue D_4_HO_2_^+^ (int) should be favored over D_4_HO_2_^+^ (ext) due to its lower ZPE, the present calculations indicate that while the D_4_HO_2_^+^ (ext) species is “invisible” at lower temperatures, it overtakes D_4_HO_2_^+^ (int) as the dominant species at 84.9 K and higher.

## Graphical abstract

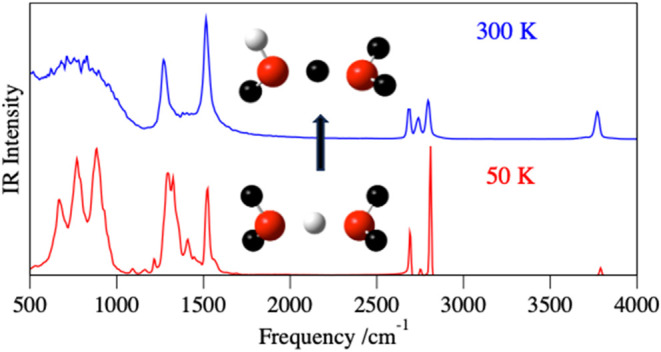

## 1 Introduction

One of the fundamental processes that is crucial for numerous areas of chemistry and biology is proton transfer (PT).^1,2^ PT occurs in acid–base reactions,^3,4^ in pathways involving renewable energy sources,^5,6^ and in atmospheric and interstellar chemistry.^7,8^ Protons in aqueous solutions, their nature and transport mechanism, and their spectroscopic signature have been a focus of intensive research for over 200 years.^9–48^ The smallest cluster where water molecules share a proton is the Zundel cation H_5_O_2_^+^.^17,18^ Due to its relatively small size, this complex was characterized using numerous quantum and classical theoretical methods on high-quality potential energy surfaces (PES),^17–35^ and accurate measurements were carried out over the past few years.^13–16,36–48^ Those studies focused on the shared PT, the H_2_O/D_2_O bend, and the OH/OD stretch vibrations in the 600–3800 cm^–1^ range. However, spectral signatures of the H_5_O_2_^+^ cluster in the far-IR or terahertz (THz) region (<600 cm^–1^), sampling specifically intermolecular stretching, torsion, wag, and rocking deformation modes, have not yet been fully understood.

THz spectroscopy advancements have created new opportunities to explore the low-frequency spectral domain and gain a deeper understanding of chemical and biological phenomena.^49^ Recently, it has become a valuable experimental tool for providing direct insights into solute–solvent interactions through hydrogen-bonding networks in aqueous solutions,^50^ and in combination with *ab initio* molecular dynamics simulations, it represents a powerful tool in the characterization of low-frequency vibrations of water in biomolecules.^51^ THz spectroscopy in connection with driven molecular dynamics (DMD)^52^ was recently used to interpret weakly absorbing IR modes of protonated water clusters, H^+^(H_2_O)_*n*=3,4_, in the 200–2200 cm^–1^ spectral range.

Another method that is widely used to characterize vibrational spectra is Raman scattering.^53–55^ Vibrational motion that changes the molecular dipole moment, i.e., asymmetric in nature, is detectable in the IR spectrum, while motion involving polarizability, often totally symmetric in nature, is active in the Raman spectrum. Therefore, examining both infrared and Raman spectra offers mutually complementary information about the molecular system. For this purpose, to achieve this goal, multidimensional PES, dipole moment surfaces (DMS), and polarizability tensor surfaces (PTS) need to be fitted preferably to high-level *ab initio* electronic structure data. While constructing analytic PES and DMS, often in global forms, is currently almost routine,^23,27^ generating similar quality PTS is far more challenging and remains in the early stages of development. Empirical models of molecular polarizability have been reported in recent years.^56–58^ Others have carried out classical simulations of Raman spectra of liquid water by utilizing density functional perturbation theory.^59^ Truncated many-body expansions of monomer interactions have been successfully used to calculate Raman spectra of liquid water using molecular dynamics (MD) simulations.^60,61^ Machine learning techniques for making efficient PTS have been tested and applied on a variety of complex systems with very good results.^62–64^ Approaches based on the classical dipole polarizability model have also been proposed and tested.^35,65,66^

Particularly intriguing are the spectra of singly deuterated H_5_O_2_^+^ species since their vibrational picture depends sensitively on the placement of the isotope, i.e., in one of the four equivalent external (ext) positions or the sole internal position (int). Previous calculations of the D_4_HO_2_^+^ species suggested that at low temperatures (50 K or less), the (int) species should be favored over the (ext) due to its substantially smaller ZPE.^23,45,47^ However, temperature effects have not been considered rigorously for H/D mixed isotopologues, and the roles of the rotational density of states and spatial entropy remain unclear. Here, we report extensive MD simulations of infrared and Raman spectra of H_5_O_2_^+^ and its singly deuterated analogs, along the fully deuterated species, D_5_O_2_^+^, using existing PES and DMS,^26^ and a recently described PTS.^35^ We focus specifically on the quantum mechanically elusive O···O stretch region, found in the [500–700] cm^–1^ frequency range, and analyze both IR and Raman spectra as functions of temperature spanning from 50 to 300 K.

## 2 Computational Methods

We carried out molecular dynamics simulations of IR and Raman spectra for H_5_O_2_^+^ and its deuterium isotopic analogs, D_5_O_2_^+^, D_4_HO_2_^+^, and DH_4_O_2_^+^. In this study, we used the full-dimensional, analytical form of the PES and DMS^26^ and a polarizability tensor surface (PTS) fitted at the CCSD­(T) level of theory, with the slightly truncated basis set of aug-cc-pVTZ quality (AVTZ-tr) (excluding *f* and *d* functions on oxygen and hydrogen, respectively).^35^ The basis set reduction resulted in an error of only 0.3% in the polarizability of H_5_O_2_^+^ evaluated at the global minimum configuration, compared to the complete aug-cc-pVTZ basis set.^35^ The fitted PTS sampled nuclear configurations up to the H_3_O^+^ + H_2_O dissociation limit (∼12,000 cm^–1^), including the low-energy isomerization transition states, the hydrogen–hydrogen exchange barrier, and high-energy stationary points reported previously by Huang et al.^26^

To establish a reference for IR and Raman intensities and to identify frequency shifts related to H/D isotopic substitution at the harmonic level, we carried out normal-mode analysis (NMA) calculations for all isotopologues. NMA was done at the MP2/aug-cc-pVTZ level of theory, using the Gaussian program^67^ and the HBB PES.^26^ For MD simulations of the spectra, we generated trajectories with randomly sampled velocities and coordinates of equilibrium geometry. Velocities were sampled from a uniform distribution corresponding to energies *k*_B_ *T* and temperatures 50, 100, 200, and 300 K (*k*_B_ = 1.38065 × 10^–23^ J.K^–1^) and propagated for 100 ps as NVE ensembles. We used the velocity-Verlet integrator with a time step of 0.2 fs. A small time step is crucial for maintaining numerical stability in the velocity-Verlet integration algorithm, enabling conservation of total energy in NVE simulations and accurate sampling of high-frequency motions, such as OH/OD stretch vibrations. It would be very expensive with a direct MD approach, but MD simulations are feasible with analytic PES/DMS/PTS as used in this work. We then time-averaged the correlation functions over the length of the trajectories using a 6.5 ps signal window to obtain smoothed signals with a Fourier resolution of ∼5 cm^–1^. IR spectra were obtained by Fourier transforming the time-averaged dipole–dipole correlation functions and scaled by a quantum mechanical factor^68^

1 $$I_{\mathrm{IR}}\left(\omega \right)=\omega /\left[1-\exp\left(-\omega /\mathrm{kT}\right)\right]\int_{0}^{\infty }dte^{-i\omega t}\left\langle \mathrm{\mu ⃗}\left(0\right)\cdot \mathrm{\mu ⃗}\left(t\right)\right\rangle$$

For calculations of Raman spectra, the polarizability tensor signal was decomposed into the isotropic α̅ ≡ (α_*xx*_ + α_*yy*_ + α_*zz*_)/3 and the anisotropic β_*ij*_ ≡ α_*ij*_ – δ_*ij*_α̅ components for *i, j = x, y, z*. Thus, the polarized and depolarized components of the Raman spectrum were calculated by the formulas^69^

2 $$I_{\mathrm{pol}}\left(\omega \right)=\omega /\left[1-\exp\left(-\omega /\mathrm{kT}\right)\right]\int_{0}^{\infty }dte^{-i\omega t}\left\langle \mathrm{\alpha ̅}\left(0\right)\mathrm{\alpha ̅}\left(t\right)\right\rangle$$

3 $$I_{\mathrm{depol}}\left(\omega \right)=\omega /\left[1-\exp\left(-\omega /\mathrm{kT}\right)\right]\int_{0}^{\infty }dte^{-i\omega t}\left\langle \sum_{i,j=x,y,z}\beta_{\mathrm{ij}}\left(0\right)\beta_{\mathrm{ij}}\left(t\right)\right\rangle$$

with the correlation function being time-averaged using the same procedural parameters as in the corresponding IR spectral calculations.

## 3 Results and Discussion

IR and Raman spectra of H_5_O_2_^+^ and its deuterium isotopologues, D_5_O_2_^+^, DH_4_O_2_^+^, and D_4_HO_2_^+^, were obtained at various temperatures. The MD simulations originated at the global minimum (Table S1) optimized on the analytical **H**_**5**_ **O**_**2**_^**+**^ **PES**.^26^ Dipole moments and polarizabilities were evaluated using the analytical **H**_**5**_ **O**_**2**_^**+**^ **DMS** ^26^ and **PTS** ^35^ and saved along the trajectory to compute IR and Raman intensities. The H_5_O_2_^+^ polarizabilities, α*_ii_* evaluated at the global minimum are listed in Table S2. To facilitate mode assignment in the vibrational spectra, we report harmonic frequencies and IR and Raman intensities for H_5_O_2_^+^ and its deuterium isotopologues (Tables S3–S8) evaluated at the MP2/aug-cc-pVTZ level of theory. The thermal averaging for the mixed H/D isotopologues DH_4_O_2_^+^ and D_4_HO_2_^+^, which comprise H and D minority species either in the internal (int) or external (ext) positions, was done using the harmonic oscillator and rigid rotor approximations.^69^ The structures denoting (int) and (ext) mixed H/D isotopologues are displayed in Scheme S1.

Here, we examine the spectra of H_5_O_2_^+^ and the other deuterium-substituted analogs, starting with the spectra calculated at 50 K. In our previous work,^34^ mixed deuterium-substituted analogs with H/D in both (int) and (ext) positions were examined separately. In this work, the IR and Raman spectra for the mixed isotopologues DH_4_O_2_^+^ and D_4_HO_2_^+^ are averaged using the Boltzmann factors P_n_(int) and P_n_(ext) described above and in Section S-2. As a general preface to the discussion that follows, we point out that the thermally averaged (int) and (ext) species are mixtures of the C_2_ and C_1_ points group symmetries, respectively, and thus in the cases where the probability of an (ext) species is not vanishingly small, the resultant spectra will contain all the vibrational transitions in both the IR and Raman cases regardless of the C_2_ point group selection rules.

The barrier height for the (int)–(ext) conversion is represented as a C_s_ structure 3944 cm^–1^ above the global minimum,^26,35^ and therefore virtually inaccessible at the temperatures considered in the present work. Relevant data, including harmonic frequencies for the HH-exchange transition state (Table S9) and ZPE corrections for this barrier, are shown in Section S-1. Due to differences in the zero-point energies (ZPE), structural degeneracy factor *g*_*i*_, and molecular symmetry σ*_i_*, the vibrational and rotational partition functions of mixed isotopologues differ substantially. Vibrational partition functions were calculated using the harmonic frequencies scaled by a ratio of harmonic zero-point energies (this work) and the diffusion Monte Carlo (DMC) zero-point energies,^45^ evaluated at the same **H**_**5**_ **O**_**2**_^**+**^ **PES** ^26^ (Table 1).

**Table 1:** Harmonic, DMC Zero-Point Energies (in cm^–1^) and Their Ratio for H_5_O_2_^+^ and Its Deuterium Isotopologues

isotopologue	harmonic ZPE^a^	DMC ZPE^b^	harmonic/DMC ratio
H_5_O_2_^+^	12,634	12,393	0.9809
D_5_O_2_^+^	9270	9143	0.9863
D_4_HO_2_^+^(int)	9795	9706	0.9909
D_4_HO_2_^+^(ext)	9987	9821	0.9833
DH_4_O_2_^+^(int)	12,116	11,837	0.9769
DH_4_O_2_^+^(ext)	11,930	11,721	0.9825

a This work.

b Reference 45.

The rotational partition function parameters for the mixed isotopologues with the minority H/D species in the internal position are *g*_*i*_ = 1 and σ*_i_* = 2, while for those in the external position, *g*_*i*_ = 4 and σ*_i_* = 1. See Section S-2 for more details on calculating the partition function. Computed Boltzmann-weighted factors for D_4_HO_2_^+^ and DH_4_O_2_^+^ as a function of temperature are shown in Figure 1.

**Figure 1:**
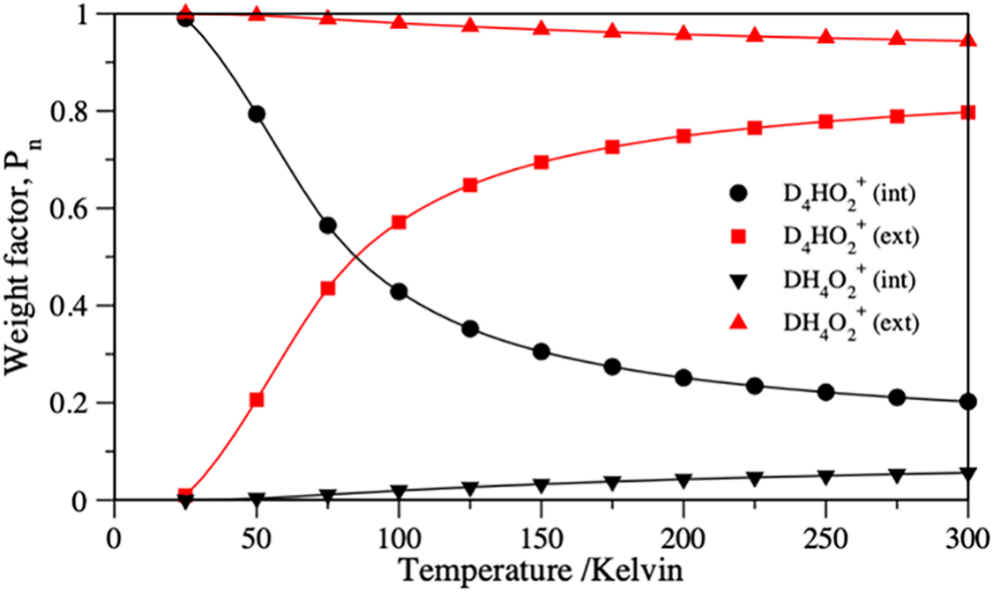
Normalized Boltzmann weight factors for the mixed isotopologues, D_4_HO_2_^+^ and DH_4_O_2_^+^, which comprise H and D minority species either in internal (black lines) or external positions (red lines). The crossing point represents the temperature of ∼84.9 K at which D_4_HO_2_^+^ (ext) overtakes D_4_HO_2_^+^ (int). Data ranging from 25 to 300 K were interpolated using cubic splines.

As described in recent works on this subject and also confirmed presently, the DH_4_O_2_^+^ (ext) isotopologue, where the D sits in the external position, dominates with a 90% probability up to the room temperature (Figure 1) and apparently much farther beyond, primarily due to the appreciably lower ZPE, i.e., having a −186 and −116 cm^–1^ difference at the harmonic and the DMC levels, respectively (see Table 1) and a 4:1 ratio of the available spatial structures. The lowest fundamental frequencies are 156 and 169 cm^–1^ for DH_4_O_2_^+^ (ext) and DH_4_O_2_^+^ (int), respectively, and are calculated using the PES (see Tables S5 and S7), which gives the preference to the (ext) isotopologue if the temperature were increased. Also, a larger rotational constant due to the 4:1 favor in the structural degeneracy factor explains a strong temperature preference for the former, which is prominently observed in Figure 1.

In a similar manner and employing the primal argument of the favorable ZPE, it was previously suggested^34,45,47^ that the mixed “heavy” isotopologue, D_4_HO_2_^+^ (int), with the H^+^ in the shared position, should be favored over its (ext) counterpart due to a comparatively large favorable ZPE differential with the (int) species, −192 and −115 cm^–1^ at the harmonic and DMC levels, respectively (see Table 1). Interestingly, our calculations show that the probability of D_4_HO_2_^+^ (ext), while extremely small at lower temperatures, rises rapidly from 25 K and on, and then at temperatures approaching ∼84.9 K, it actually overtakes that of the D_4_HO_2_^+^ (int) species. A detailed analysis reveals that the lowest vibrational fundamental frequency, being 131 cm^–1^ for (ext) and 121 cm^–1^ for (int) (see Tables S6 and S8), should give a slight preference to the latter if the temperature were increased. Along with it, a smaller rotational constant for the D_4_HO_2_^+^ (int) suggests a more preferable configuration, yet the spatial degeneracy factor of 4:1 for (ext) over (int) multiplied by the C_2_ point group symmetry factor of 2 entering the rotational partition function eventually helps the (ext) structure “win” at what we call a critical temperature, 84.9 K. Above the critical temperature, the D_4_HO_2_^+^ (ext) species rapidly becomes more viable. With the statistical basis having been laid out beforehand, we will use it below in the thermal averaging of the IR and Raman spectra. The IR spectra of the mixed isotopologues, D_4_HO_2_^+^ (Figure 2) and DH_4_O_2_^+^ (Figure 3), agree remarkably well with the previously published experimental measurements.^48^

**Figure 2:**
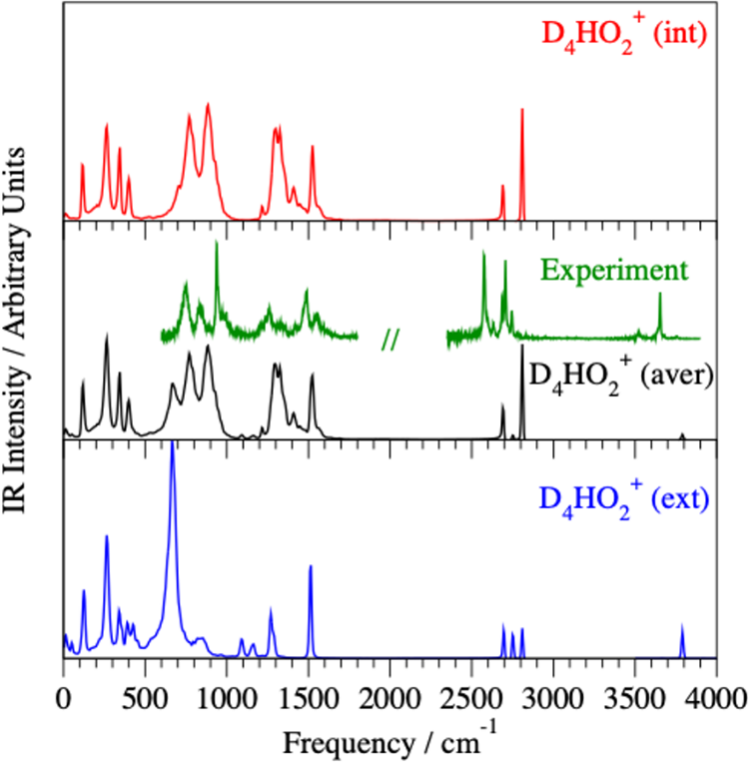
Simulated IR spectra for the H minority species in the internal (red line) and external (blue line) positions are compared to the averaged D_4_HO_2_^+^ spectrum (black line) calculated at energies corresponding to 50 K. The corresponding D_4_HO_2_^+^·Ar predissociation experimental spectra^48^ are shown as green lines.

**Figure 3:**
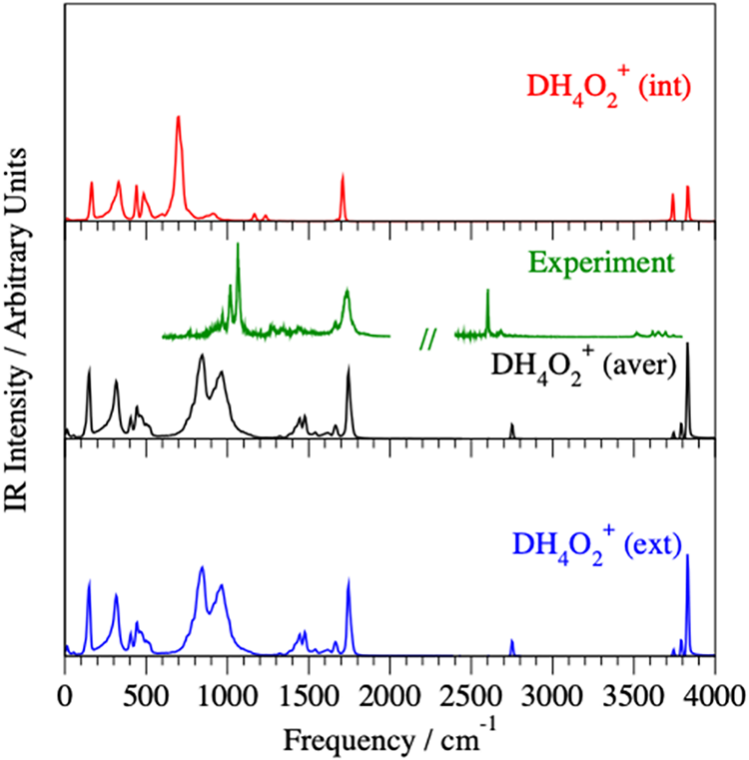
Simulated IR spectra for the D minority species in the internal (red line) and external (blue line) positions are compared to the averaged DH_4_O_2_^+^ spectrum (black line) calculated at energies corresponding to 50 K. The corresponding DH_4_O_2_^+^·Ar predissociation experimental spectra^48^ are shown as green lines.

To the best of our knowledge, there are no reported Raman spectra of a protonated water cluster, H_5_O_2_^+^, and its deuterium-substituted analogs in the literature calculated beyond the double harmonic approximation. Our classical MD Raman spectra of H_5_O_2_^+^ and deuterium isotopologues are described for the first time and complement numerous theoretical studies, including our previous work,^33,34^ quantum studies,^17–22^ and experimental measurements.^45,48^ The polarized and depolarized components of the Raman spectra (eqs 2 and 3) are shown side by side with the IR spectra (eq 1) in Figure 4 (high-frequency range) and Figure 5 (low-frequency range). The most intense Raman activities correspond to OH/OD stretch vibrations, and the weak activities are observed in the low-frequency range (see also the Raman spectra in the full range, Figure 1S). High-quality Raman spectra of liquid water were described by Medders and Paesani,^61^ and they have shown patterns similar to our protonated water dimer spectra with an intense peak in the OH stretch range.

**Figure 4:**
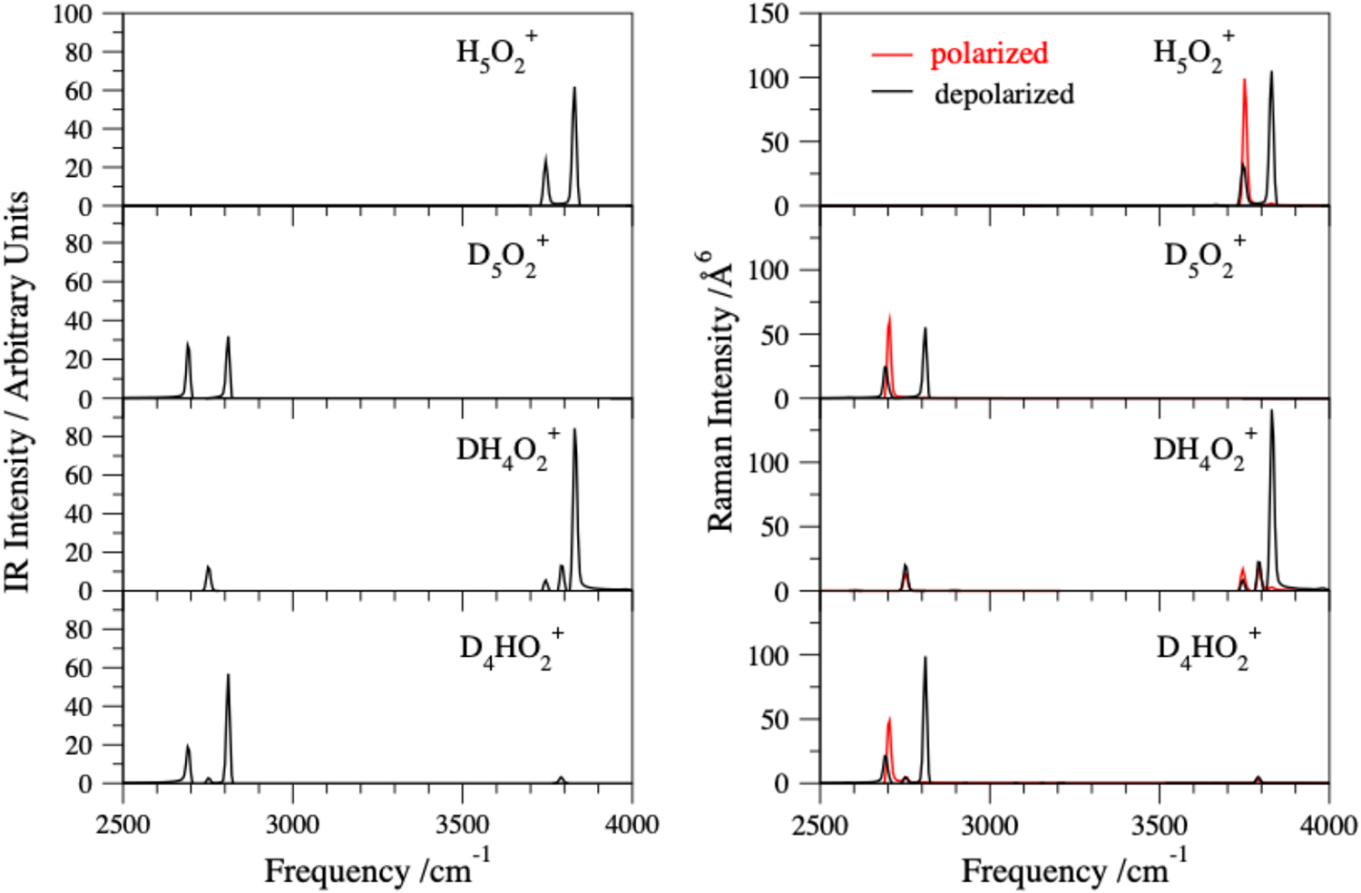
Simulated IR spectra (left panel) and Raman spectra (right panel) in the high-frequency range of H_5_O_2_^+^ and its deuterium isotopologues were calculated at the energies corresponding to 50 K. The IR and Raman intensities for DH_4_O_2_^+^ and D_4_HO_2_^+^ mixed isotopologues with H or D minority species in the internal and external positions were averaged using the Boltzmann factors P_n_(int) and P_n_(ext).

**Figure 5:**
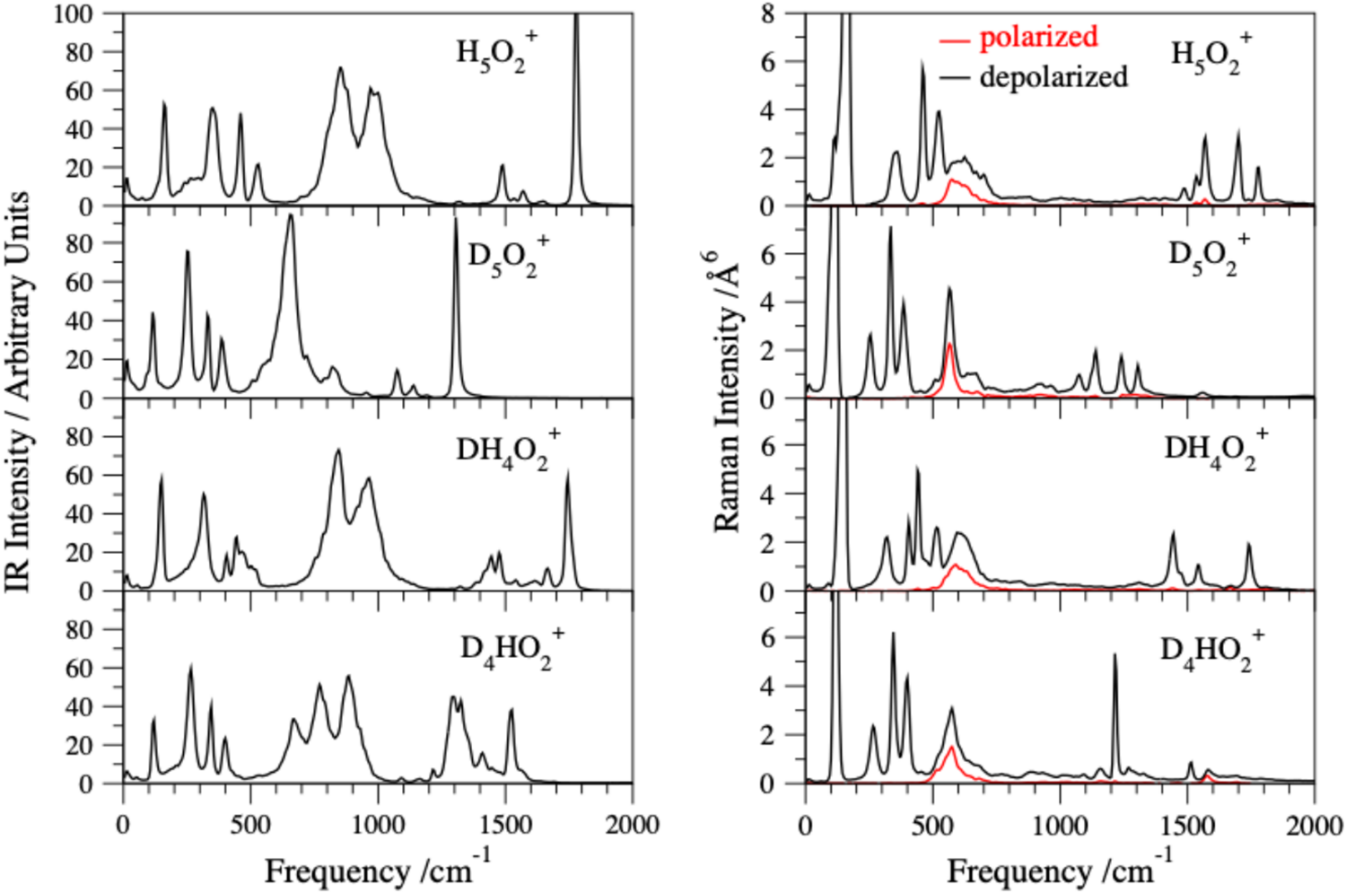
Simulated IR spectra (left panel) and Raman spectra (right panel) of H_5_O_2_^+^ and its deuterium isotopologues were calculated at the energies corresponding to 50 K. The IR and Raman intensities for DH_4_O_2_^+^ and D_4_HO_2_^+^ mixed isotopologues with H or D minority species in the internal and external positions were averaged using the Boltzmann factors P_n_(int) and P_n_(ext).

The H_5_O_2_^+^ Raman spectrum in the higher frequency range (Figure 4) shows an intense, sharp peak at 3750 cm^–1^, which is ascribed to the symmetric in-phase OH stretch vibration. Interestingly, the polarized and depolarized Raman spectra in the high-frequency range clearly distinguish between the two symmetric in-phase OH (3750 cm^–1^) and out-of-phase OH stretches (3745 cm^–1^) separated by only a few wavenumbers yet within the resolution of the simulated spectra. For a zoomed-in plot, see Figure 2S. The other peak of the doublet at 3830 cm^–1^ corresponds to two closely overlapping asymmetric in-phase and out-of-phase OH stretch vibrations.

In the D_5_O_2_^+^ Raman spectrum (Figure 4), we can further discern that the OD stretch is generally red-shifted by about 1000 cm^–1^ compared to the respective OH stretch in the H_5_O_2_^+^ spectrum. Similar to H_5_O_2_^+^, the two symmetric in-phase OD (2705 cm^–1^) and out-of-phase stretches (2690 cm^–1^) are well-resolved in the high-frequency range of the D_5_O_2_^+^-polarized/depolarized Raman spectra (Figure 3S).

At 50 K, the thermally averaged IR and Raman spectra in the high-frequency range for the mixed isotopologues, DH_4_O_2_^+^ and D_4_HO_2_^+^ (Figure 4), show four in-phase/out-of-phase OH/OD stretch peaks at the corresponding frequencies that arise due to the broken symmetry introduced by the H/D substitution. Our calculations overestimate the OH/OD stretch frequencies compared to experimental measurements (Figures 2 and 3). OH and OD reduced masses are relatively light, which makes their vibrational frequency theoretical predictions particularly sensitive to the accuracy of trajectory propagation and temperature. At a low temperature, the MD spectra reproduce the harmonic spectra. As the temperature rises, classical spectra differ from harmonic spectra because of the classical response to anharmonic effects.^34^ OH/OD stretch frequencies were slightly red-shifted with the temperature, as seen in Figure 5S. The IR experimental spectra show additional peaks due to the presence of the Ar tag.^45,48^

The polarized and depolarized components of the Raman spectra of H_5_O_2_^+^ and its deuterium isotopologues at 50 K provide additional information about symmetry-related vibrations. As there is no experiment in the low-frequency range, our assignment is based on normal-mode analysis and the published quantum studies.^19,21,24^ The low-frequency range of the IR spectrum and the depolarized Raman spectrum of H_5_O_2_^+^ (Figure 5, black line) correspond to torsion (170 cm^–1^), wagging (350, 460 cm^–1^), and two overlapping rocking (525 cm^–1^) fundamentals. The broad activity in the depolarized Raman spectrum at [500–700] cm^–1^ is identified as the O···O symmetric stretch that involves intermolecular rotational symmetry-breaking motions. Furthermore, the H_5_O_2_^+^-polarized Raman spectrum (Figure 5, red line) exhibits a broad feature that originates near 550 cm^–1^ and extends to ∼700 cm^–1^, corresponding to the O···O vibration that formally preserves rotational symmetry about the *C*_*2*_ axis.

The IR spectra of H_5_O_2_^+^, D_5_O_2_^+^, and DH_4_O_2_^+^ at 50 K (Figure 5) show doublets in the [650–1000] cm^–1^ range that arise from a resonance between the shared proton stretch and a combination band involving one quantum of the O···O stretch and two quanta of the water wag. These doublets were characterized by quantum studies conducted by Vendrell et al.^19,21^ and discussed in our previous work as red-shifted compared to the experiment.^34^ The DH_4_O_2_^+^·Ar predissociation IR experimental spectrum^48^ (Figure 3) also shows a doublet at 1020/1065 cm^–1^ and an extra weak feature in the shared proton range due to the presence of the Ar tag. The thermally averaged IR spectrum of D_4_HO_2_^+^ consists of both (int) and (ext) isotopologues at 50 K and therefore shows a triplet consisting of both H^+^/D^+^ shared doublets, similar to the experimental study^48^ (Figure 2).

In the [1450–1800] cm^–1^ bending range, there is activity in both the IR and Raman spectra. The depolarized H_5_O_2_^+^ Raman spectrum (Figure 5, black line) is more convoluted than that of its polarized counterpart (Figure 5, red line). It exhibits a structured line shape, which we presently think corresponds to H^+^ motion perpendicular to the O–O axis at 1490 cm^–1^ and two H_2_O bending modes in-phase and out-of-phase at 1710 and 1780 cm^–1^, respectively. Note that the H_2_O in-phase bending mode is dark in the IR spectrum. The presence of both polarized and depolarized components near the (1550, 1580) cm^–1^ doublet is similarly explained by a Fermi resonance between the other shared H^+^ perpendicular bend component and a combination of a water wag and two quanta of the intermolecular O···O symmetric stretch mode, in line with the previous assignment by Vendrell et al.^21^ For comparison, the corresponding deuterium-substituted isotopic bending modes in the D_5_O_2_^+^ Raman spectrum are at 1080 and 1145 cm^–1^ (shared D^+^ perpendicular bend) and 1250 and 1315 cm^–1^ (D_2_O bend), respectively.

The peaks in the DH_4_O_2_^+^ depolarized thermally averaged Raman spectrum at 1450 cm^–1^, 1490 cm^–1^, and 1750 cm^–1^ are assigned to the HDO, shared H^+^ perpendicular bend, and H_2_O bends, respectively. Another weak spectral feature in the Raman spectrum (Figure 5) at 1670 cm^–1^ corresponds to the other component of the shared H^+^ perpendicular bending vibration, while it is stronger in the IR spectrum.

The corresponding DH_4_O_2_^+^ harmonic frequencies for the H_2_O and HDO bending modes are 1449 and 1740 cm^–1^, and the shared H^+^ perpendicular bending modes are 1487 and 1663 cm^–1^, respectively (see Table S7). The DH_4_O_2_^+^·Ar predissociation IR experimental spectrum (Figure 3) supports our simulations, showing strong activity at 1740 cm^–1^, corresponding to the H_2_O bend mode, and weak activity at 1650 cm^–1^, corresponding to the shared H^+^ perpendicular bending mode, while HDO bending and another component of H^+^ perpendicular bending are present but relatively weak.

The anharmonic feature at 1550 cm^–1^ in the DH_4_O_2_^+^ Raman spectrum (Figures 5 and 4S) can be assigned to a Fermi resonance between the shared H^+^ perpendicular bend component and a combination of water wag and two quanta of the intermolecular O···O symmetric stretch mode,^21^ similar to the H_5_O_2_^+^ spectrum (see discussion of the H_5_O_2_^+^ Raman spectrum above). The assignment of D_4_HO_2_^+^ bending modes is temperature-dependent due to the mixing of both (int) and (ext) isotopologues and is discussed below.

The DH_4_O_2_^+^ (ext) isotopologue with an H^+^ in the shared position dominates over 90%, and the corresponding line shape is barely temperature-dependent, at least up to room temperature. The thermally averaged IR and Raman spectra of D_4_HO_2_^+^, on the other hand, strongly depend on the temperature due to heavy mixing of the (int) and (ext) conformations (Figure 1). Continuing the discussion, the D_4_HO_2_^+^ Raman spectrum at the low temperature, 50 K, can be regarded as a combination of the internal and external minority H/D species with the Boltzmann weights, P_n_(int)=0.79 and P_n_(ext)=0.21, respectively. Indeed, we observe the signatures of both isotopologues with the H in the int and ext positions (Figure 6). The high-frequency range of D_4_HO_2_^+^ (int) resembles the D_5_O_2_^+^ spectrum (Figure 4) with 2700 and 2800 cm^–1^ spectral features assigned to the symmetric OD stretch and the asymmetric OD stretch, respectively. However, the spectrum shows two additional peaks at 2755 and 3800 cm^–1^ (OD stretch and OH stretch), which are presently assigned to the ∼21% presence of the D_4_HO_2_^+^ (ext) isotopologue. Similar trends can be observed in the D_4_HO_2_^+^ IR spectra (Figure 2), which are consistent with the experimental D_4_HO_2_^+^ spectra.^48^

**Figure 6:**
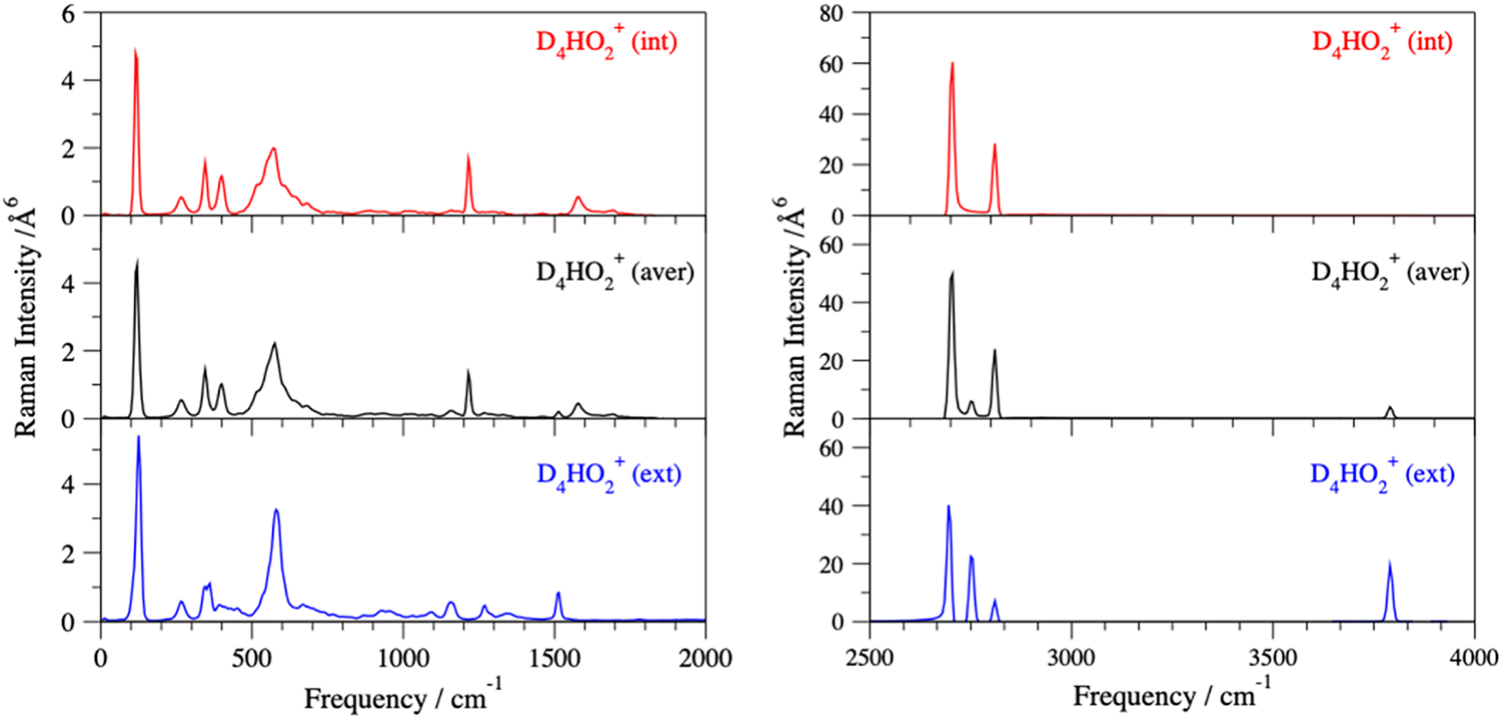
Low-frequency range (left panel) and high-frequency range (right panel) of the simulated Raman spectra showing the total Raman intensity, for the H minority species in the internal (red line) and external (blue line) positions are compared to the averaged D_4_HO_2_^+^ spectrum (black line) calculated at the energies corresponding to 50 K.

IR and Raman spectra (Figures 7 and 5S) of D_4_HO_2_^+^ show strong mixing of both (int) and (ext) isotopologues as the temperature rises from 50 to 300 K. Notably, the averaged IR spectrum of D_4_HO_2_^+^ at 50 K exhibits a rather prominent activity seen as a triplet already mentioned above (Figure 2). The peak at 680 cm^–1^ corresponds to the lower component of the shared D^+^ doublet due to the 21% presence of the D_4_HO_2_^+^ (ext) isotopologue, even at 50 K, which is otherwise missing in the pure (int) spectrum (Figure 2). This peak persists over a temperature range of 50–300 K. The second and third peaks at 770 cm^–1^ and 900 cm^–1^ are assigned to the shared H^+^ parallel stretch doublet due to the D_4_HO_2_^+^ (int) isotopologue being dominant at low temperature. Note that these two peaks disappear as the temperature rises, and the D_4_HO_2_^+^ (ext) isotopologue dominates (Figure 7).

**Figure 7:**
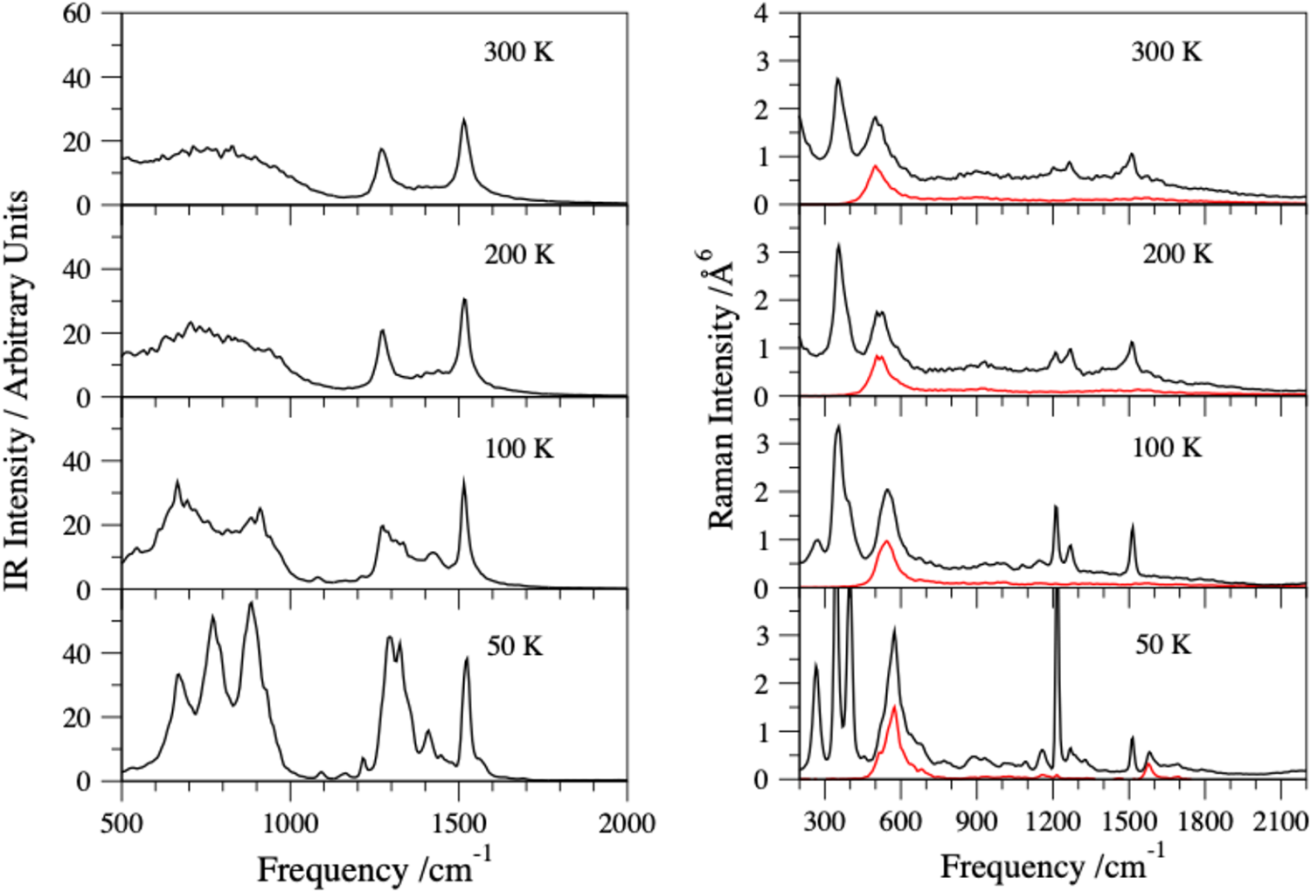
IR spectra (left panel) and Raman-polarized (red line) and depolarized (black line) spectra (right panel) of the D_4_HO_2_^+^ deuterium isotopologue calculated at energies corresponding to the temperatures from 50 to 300 K.

The intermolecular O···O stretching vibration is dark in the IR regime. It is, however, Raman-active, although with certain caveats resulting from the *C*_*2*_ symmetry-breaking at higher temperatures (Figure 7). The O···O stretch is particularly sensitive to temperature changes in the D_4_HO_2_^+^ isotopologue, as indicated by the peak in the polarized Raman signal shown in Figure 7, which occurs between 500 and 575 cm^–1^. In D_4_HO_2_^+^, the position of the proton varies with temperature: at lower temperatures, it occupies the shared position [D_2_O···H^+^···D_2_O], while at higher temperatures, it moves to the external position [HDO···D^+^···D_2_O], as illustrated by the Boltzmann weight factor plot in Figure 1.

Also, the O···O symmetric stretch frequency shows peculiar patterns in the Raman spectra for H/D isotopic substituted analogs (Figure 5), for isotopic analogs it is in the range from 565 to 590 cm^–1^, being the smallest for fully deuterated isopologue and largest for DH_4_O_2_^+^ (see Table 2). The state-of-the-art MCTDH quantum calculations by Vendrell et al.^19^ predicted the O···O stretch in H_5_O_2_^+^ at 550 cm^–1^. This was largely confirmed by our MD simulations, in part validating the high quality of the HBB PES. In the absence of exact quantum calculations and comparing the harmonic data, the MD Raman spectra revealed anharmonic shifts for the O···O intermolecular stretch vibration upon H/D substitution.

**Table 2:** Vibrational Frequencies (in cm^–1^) for the O···O Stretch Derived from the Harmonic Analysis Using the HBB PES and from the Anharmonic Calculations Derived from MD Raman Simulations at 50 K with the HBB PES and CCSD­(T)/aug-cc-pVTZ (O: *spd*, H: *sp*) PTS Reported Here

species	harmonic	MD Raman	anharmonicity
H_5_O_2_^+^	628	575	53
D_5_O_2_^+^	593	565	28
DH_4_O_2_^+^	626	595	31
D_4_HO_2_^+^	593	575	18

The assignment of the [1200–1600] cm^–1^ spectral range of D_4_HO_2_^+^ is more challenging (Figures 2, 6, and 7) due to the mixing of both internal and external isotopologues. This region contains shared H^+^ and D^+^ perpendicular bend and D_2_O and HDO bend vibrations (Tables S6 and S8). Weak activity in the IR spectrum at 1100 and 1180 cm^–1^ is assigned to the D^+^ perpendicular bend due to the contribution of D_4_HO_2_^+^(ext) to the averaged IR spectrum of D_4_HO_2_^+^. The Raman spectrum shows a strong peak at 1220 cm^–1^ that corresponds to the in-phase D_2_O bends, being dark in the IR spectrum, while the IR spectrum shows the out-of-phase D_2_O bend at 1300 cm^–1^, which is dark in the Raman spectrum. Weak activity in the IR spectrum (Figure 2) at 1435 cm^–1^ is tentatively assigned to a combination band consisting of the shared H^+^ parallel stretch (O···H^+^···O) plus intermolecular O···O stretch, based on the quantum isotopic study by Vendrell et al.^17^ At 50 K, the strong IR activity at 1510 cm^–1^ in the averaged D_4_HO_2_^+^ spectrum (Figure 2) is most likely a combination of two overlapping peaks, a shared H^+^ perpendicular bend and HDO bend belonging to (int) and (ext) components, respectively. There is weak IR activity at 1580 cm^–1^ (Figure 6) assigned to the shared H^+^ perpendicular bend. The mixing of the two D_4_HO_2_^+^ isotopologues (int) and (ext) with the H minority species in the internal and external positions, respectively, can be monitored as the temperature rises from 50 to 300 K (Figure 7). At 50 K, the Raman depolarized spectrum shows two peaks at 1510 and 1580 cm^–1^, assigned to the HDO bend and the H^+^ perpendicular bend. The presence of the peak at 1510 cm^–1^ and the absence of the 1580 cm^–1^ peak at temperatures of 100 K and above confirms the dominance of the D_4_HO_2_^+^ (ext) isotopologue as the temperature increases.

## 4 Conclusions

In this study, we have presented IR and Raman vibrational spectra of H_5_O_2_^+^ and its various deuterium-substituted isotopologues using MD simulations at a temperature range of 50–300 K. Our Raman spectra of the protonated water dimer, the first of its kind, complement the available IR theoretical and experimental studies. Utilizing the well-established HBB potential and our recently developed polarizability tensor surface, we elucidated the symmetric intermolecular O···O stretch vibration within the [500–700] cm^–1^ range, providing significant insights into the behavior of these species as a function of temperature. We also provide new insights into the H/D isotopic substitution, where DH_4_O_2_^+^ (ext) appears as the predominant species at temperatures from 50 to 300 K, primarily due to its low zero-point energy and challenging previous assertions regarding the stability of the mixed isotopologue D_4_HO_2_^+^ in the internal position. Notably, the transition point at 84.9 K, where D_4_HO_2_^+^ (ext) overtakes D_4_HO_2_^+^ (int), emphasizes the importance of thermal effects in determining the stability and distribution of isotopologues in this system. Thermally averaged spectra of partially deuterated isotopologues DH_4_O_2_^+^ and D_4_HO_2_^+^, obtained with internal energies corresponding to a temperature of 50 K, are in overall good agreement with those from experimental measurements. Our results highlight the complexity of vibrational spectra in partially deuterated species and emphasize the need to explore symmetric vibrations using Raman spectroscopy.

This work is important in advancing our understanding of the spectral characteristics of H_5_O_2_^+^ clusters and also elucidates the role of hydrogen bonding and vibrational dynamics in chemical and biological systems. Our theoretical work can be directly correlated with experimental observations and extended to larger size clusters to predict their vibrational behavior.

## Supplementary Material



## Notes

Structural parameters; polarizability; harmonic vibrational frequencies; details on the calculation of the partition function used in the thermal averaging procedure; and additional IR and Raman spectra of H_5_O_2_^+^ and its deuterium-substituted analogs (PDF)

## †

HealthTrackRx, 1500 Interstate 35W, Denton, TX 76207, email: Omodemioluwaseun@gmail.com
