# Supplementary material for: First-Principles Molecular Dynamics Simulations of Infrared and Raman Vibrational Spectra of H5O2 +, D5O2 +, DH4O2 +, and D4HO2 + from 50 to 300 K
Source: J Phys Chem A. 2025 Oct 22;129(43):9921–9. doi: 10.1021/acs.jpca.5c05184 (PMC12581142; doi:10.1021/acs.jpca.5c05184)
Supplement: Supplementary file 1 [file jp5c05184_si_001.pdf]

First Principles Molecular Dynamics Simulations of Infrared and Raman Vibrational Spectra of $H_5O_2^+$, $D_5O_2^+$, $DH_4O_2^+$, and $D_4HO_2^+$ from 50 to 300 Kelvin

Supporting Information

*Oluwaseun Omodemi, Martina Kaledin**

Department of Chemistry & Biochemistry, Kennesaw State University, 370 Paulding Ave NW, Box # 1203, Kennesaw, Georgia 30144

Table of contents

S-1 Structure, energetics, and vibrational frequencies of $H_5O_2^+$ and its deuterium-substituted analogs……………………………………………………………………………………S2

S-2 Calculation of the partition function …………………………………………………...S10

S-3 Additional IR and Raman spectra of $H_5O_2^+$ and its deuterium-substituted analogs……S12

References ……………………………………………………………………………….S16

Corresponding Author

*Email: Martina Kaledin, mkaledin@kennesaw.edu

S-1 Structure, energetics, and vibrational frequencies of $H_5O_2^+$ and its deuterium-substituted analogs

This section includes the $H_5O_2^+$ XYZ equilibrium coordinates (**Table S1**) used for all MD simulations with the analytical potential energy surface,[1] **$H_5O_2^+$ HBB PES**. The molecular polarizabilities of $H_5O_2^+$ calculated in this work, using the analytical **$H_5O_2^+$ PTS** at the equilibrium geometry, are listed in **Table S2**. Harmonic vibrational frequencies for $H_5O_2^+$ and its deuterium isotopologues calculated with the analytical **$H_5O_2^+$ PES,** along with the corresponding harmonic frequencies, IR and Raman intensities computed using MP2 and CCSD(T) methods with the aug-cc-pVTZ basis set are provided in **Tables S3-S8**. Additionally, we present data for the HH and HD exchange transition states (**Table S9**) to assess the barrier height for the interconversion of the (int) and (ext) H/D mixed isotopologues.

Table S1: XYZ coordinates in Å for the $H_5O_2^+$ global minimum optimized on the analytical $H_5O_2^+$ PES (CCSD(T)/aug-cc-pVTZ) level of theory,[1] (C_2 symmetry)

Atom	X	Y	Z
O	1.19322478184551	0.000000000000000	0.000000000000000
O	-1.19322478184551	0.000000000000000	0.000000000000000
H	0.00000000000000	0.000000000000000	0.06535885965756268
H	-1.59451397048087	0.788042120022264	-0.395082646428001
H	-1.69420716958714	-0.240501029111350	0.792779623059018
H	1.59451397048087	-0.788042120022264	-0.395082646428001
H	1.69420716958714	0.240501029111350	0.792779623059018

Table S2: Polarizability, α_{ii} data in atomic units for $H_5O_2^+$ evaluated using analytical $H_5O_2^+$ PTS at the geometry listed in Table S1

	X	Y	Z
X	20.1156457976	-0.7512603978	0.0000000000
Y	-0.7512603978	13.9879702954	0.0000000000
Z	0.0000000000	0.0000000000	14.0132665782

Table S3: Harmonic frequencies (in cm^{-1}) for the $H_5O_2^+$ Zundel complex. *Ab initio* data (MP2 and CCSD(T) with the aug-cc-pVTZ basis set) were compared to the analytical potential energy surface fit (HBB PES).[1] MP2 frequencies, IR intensities (in km/mol), and Raman intensities (Å^4/amu) were calculated using the Gaussian program,[2] while CCSD(T) frequencies and IR intensities were calculated using the MOLPRO program[3]

Method			MP2	MP2	MP2	CCSD(T)	CCSD(T)	HBB
Mode	Assignment	Labels	freq.	IR int.	Raman int.	freq.	IR int.	PES freq.
1	torsion	A	171	40.4	0.7	174	39.0	169
2	wagging	B	367	275.5	0.2	337	390.4	337
3	wagging	A	462	130.4	0.6	465	123.0	471
4	rocking	B	535	74.2	0.2	534	116.8	531
5	rocking	A	536	60.3	0.2	542	64.7	554
6	O···O stretch	A	624	0.0	4.5	626	0.0	628
7	H^+ transfer paral.	B	911	3025.5	0.4	850	3011.8	861
8	H^+ transfer perp.	B	1473	267.7	0.5	1482	251.4	1494
9	H^+ transfer perp.	A	1550	98.2	2.3	1562	99.7	1574
10	H_2O bend, in phase	A	1706	2.6	0.7	1719	2.4	1720
11	H_2O bend, out of phase	B	1761	963.0	0.6	1769	882.5	1770
12	OH sym.stretch, out of phase	B	3733	241.0	9.8	3740	218.4	3744
13	OH sym. stretch, in phase	A	3741	8.9	134.8	3747	7.5	3751
14	OH asym. stretch	A	3837	240.7	15.9	3832	216.6	3832
15	OH asym. stretch	B	3838	336.5	17.0	3833	305.2	3832

Table S4: **Harmonic analysis for the $D_5O_2^+$ isotopologue. See Table S3 for the method description**

Method			MP2	MP2	MP2	CCSD(T)	CCSD(T)	HBB
Mode	Assignment	Labels	freq.	IR int.	Raman int.	freq.	IR int.	PES freq.
1	torsion	A	122	18.5	0.4	125	18.1	121
2	wagging	B	270	143.0	0.1	248	213.2	247
3	wagging	A	334	52.2	0.3	337	50.0	339
4	rocking	B	392	65.0	0.2	391	96.3	389
5	rocking	A	405	39.5	0.1	411	40.5	420
6	O···O stretch	A	588	2.8	4.1	591	3.0	593
7	D^+ transfer paral.	B	660	1469.9	0.2	623	1454.9	627
8	D^+ transfer perp.	B	1069	106.2	0.3	1080	101.9	1084
9	D^+ transfer perp.	A	1127	45.4	0.8	1139	46.5	1145
10	D_2O bend, in phase	A	1248	0.2	0.7	1261	0.2	1257
11	D_2O bend, out of phase	B	1292	553.0	0.3	1301	512.1	1298
12	OD sym. stretch, out of phase	B	2687	154.9	5.0	2701	141.9	2694
13	OD sym. stretch, in phase	A	2698	6.1	67.0	2711	5.0	2703
14	OD asym. stretch	A	2817	126.7	7.9	2822	54.0	2811
15	OD asym. stretch	B	2817	174.7	8.7	2822	159.1	2811

Table S5: **Harmonic analysis for the $DH_4O_2^+$ (int) isotopologue with the minority species in the internal position. See Table S3 for the method description**

Method			MP2	MP2	MP2	CCSD(T)	CCSD(T)	HBB
Mode	Assignment	Labels	freq.	IR int.	Raman int.	freq.	IR int.	PES freq.
1	torsion	A	171	40.3	0.7	175	39.4	169
2	wagging	B	343	280.7	0.2	308	392.8	311
3	wagging	A	441	88.7	0.5	444	84.7	444
4	rocking	B	494	122.8	0.2	493	170.2	488
5	rocking	A	524	101.8	0.2	533	104.2	547
6	O···O stretch	A	622	0.0	4.5	625	0.0	626
7	D^+ transfer paral.	B	720	1618.3	0.3	693	1515.1	692
8	D^+ transfer perp.	B	1157	75.3	0.4	1168	76.3	1173
9	D^+ transfer perp.	A	1226	60.8	1.0	1239	61.9	1244
10	H_2O bend, in phase	A	1660	12.6	1.6	1679	12.8	1675
11	H_2O bend, out of phase	B	1693	518.1	0.9	1711	500.6	1707
12	OH sym. stretch, out of phase	B	3733	271.2	9.9	3753	249.4	3743
13	OH sym. stretch, in phase	A	3741	9.2	135.0	3761	7.8	3750
14	OH asym. stretch	A	3837	240.6	15.9	3846	218.2	3831
15	OH asym. stretch	B	3838	336.5	17.0	3847	307.7	3832

Table S6: **Harmonic analysis for the $D_4HO_2^+$ (int) isotopologue with the minority species in the internal position. See Table S3 for the method description**

Method			MP2	MP2	MP2	CCSD(T)	CCSD(T)	HBB
Mode	Assignment	Labels	freq.	IR int.	Raman int.	freq.	IR int.	PES freq.
1	torsion	A	122	18.4	0.4	125	18.1	121
2	wagging	B	281	139.9	0.1	263	207.3	261
3	wagging	A	344	61.6	0.3	347	58.9	350
4	rocking	B	409	44.4	0.2	410	68.4	407
5	rocking	A	410	29.8	0.1	416	31.3	423
6	O···O stretch	A	588	2.7	4.1	591	3.0	593
7	H^+ transfer paral.	B	831	2204.1	0.4	777	2458.7	786
8	H^+ transfer perp.	B	1514	410.5	0.04	1525	333.6	1522
9	H^+ transfer perp.	A	1565	52.7	1.31	1582	54.2	1584
10	D_2O bend, in phase	A	1205	19.3	0.8	1219	19.6	1219
11	D_2O bend, out of phase	B	1299	1527.3	0.5	1297	1343.2	1303
12	OD sym. stretch, out of phase	B	2689	125.6	5.0	2702	113.0	2695
13	OD sym. stretch, in phase	A	2698	5.7	66.8	2711	5.2	2703
14	OD asym. stretch	A	2817	127.9	7.9	2822	115.9	2811
15	OD asym. stretch	B	2818	176.3	8.7	2822	160.5	2812

Table S7: **Harmonic analysis for the $DH_4O_2^+$ (ext) isotopologue with the minority species in the external position. See Table S3 for the method description**

Method		MP2	MP2	MP2	CCSD(T)	CCSD(T)	HBB
Mode	Assignment	freq.	IR int.	Raman int.	freq.	IR int.	PES freq.
1	torsion	157	35.2	0.6	161	34.8	156
2	wagging	329	198.2	0.2	308	295.6	306
3	wagging	444	80.7	0.6	447	73.2	447
4	rocking	470	146.4	0.1	470	189.2	482
5	rocking	530	44.4	0.2	535	62.4	534
6	O···O stretch	622	0.3	4.5	624	0.5	627
7	H^+ transfer paral.	896	2888.8	0.5	837	2937.7	846
8	H^+ transfer perp.	1466	235.4	0.5	1481	212.4	1487
9	H^+ transfer perp.	1648	305.6	0.8	1664	302.1	1663
10	HDO bend	1432	303.6	1.7	1446	292.2	1449
11	H_2O bend	1729	591.4	0.8	1745	513.7	1740
12	OD stretch	2750	102.3	23.9	2759	93.0	2752
13	OH stretch	3737	127.2	68.8	3757	116.1	3747
14	OH stretch	3794	209.6	46.1	3808	189.7	3794
15	OH stretch	3838	289.2	16.4	3846	263.7	3832

Table S8: **Harmonic analysis for the $D_4HO_2^+$ (ext) isotopologue with the minority species in the external position. See Table S3 for the method description**

Method		MP2	MP2	MP2	CCSD(T)	CCSD(T)	HBB
Mode	Assignment	freq.	IR int.	Raman int.	freq.	IR int.	PES freq.
1	torsion	132	22.9	0.4	135	22.5	131
2	wagging	286	159.3	0.1	262	241.5	263
3	wagging	344	57.7	0.3	347	56.1	347
4	rocking	399	77.7	0.2	399	102.7	398
5	rocking	473	65.2	0.2	480	64.0	487
6	O···O stretch	595	11.6	4.1	597	35.8	598
7	D^+ transfer paral.	676	1523.9	0.3	640	1472.3	644
8	D^+ transfer perp.	1080	103.7	0.3	1091	100.4	1095
9	D^+ transfer perp.	1158	84.2	0.9	1169	80.7	1173
10	D_2O bend	1265	170.1	0.5	1277	160.0	1273
11	HDO bend	1499	328.2	0.8	1515	309.1	1512
12	OD stretch	2692	84.9	33.2	2705	77.8	2698
13	OD stretch	2756	103.1	27.2	2765	94.1	2755
14	OD stretch	2817	151.0	8.3	2822	137.3	2811
15	OH stretch	3786	224.3	42.1	3800	204.6	3789

Table S9: Energies (cm^{-1}), harmonic frequencies and zero-point energies (ZPE) (in cm^{-1}) for the $H_5O_2^+$ Zundel complex: HH and HD exchange transition states calculated using the analytical potential energy surface fit (HBB PES)[1]

Transition state	$H_5O_2^+$	$DH_4O_2^+$	$D_4HO_2^+$
1	496i	418i	403i
2	238	229	181
3	311	306	272
4	366	347	286
5	413	409	314
6	450	449	345
7	1140	1025	962
8	1597	1367	1239
9	1650	1611	1255
10	1710	1709	1439
11	3552	2616	2586
12	3626	3587	2727
13	3754	3750	2762
14	3816	3811	2826
15	3860	3860	3607
ZPE	13241	12538	10400

Based on optimized energies and the ZPE values (main text Table 1 and Table S9), the $H_5O_2^+$ minimum (0.0) and C_s-HH exchange transition state (0.01796958 hartrees = 3944 cm^{-1}, the ZPE corrected barrier height for HH exchange is 4552 cm^{-1}. The ZPE corrected barrier heights for the HD exchange, relative to the $DH_4O_2^+$ TS are 4366 and 4552 cm^{-1} for the $DH_4O_2^+$ (int) and $DH_4O_2^+$ (ext), respectively. The ZPE corrected barrier heights for the DH exchange, relative to the $D_4HO_2^+$ TS are 4549 and 4357 cm^{-1} for the $D_4HO_2^+$ (int) and $D_4HO_2^+$ (ext), respectively.

S-2 Calculation of the partition function

The total partition function, Z_n for the species $D_nH_{5-n}O_2^+$ with n = 0, 1, 4, 5, is a product of individual factors,[4]

$$Z_n = gZ_{rot}Z_{vib}exp\left(-\frac{\Delta E_n}{kT}\right) \qquad (S1)$$

where g is the species degeneracy factor (see **Scheme S1**).

Z_{rot} is the rigid rotor rotational partition function with the moments of inertia I_a, I_b, I_c and the symmetry factor σ (see **Scheme S1**),

$$Z_{rot} = \frac{1}{\sigma}(kT)^{\frac{3}{2}}(8\pi I_a I_b I_c)^{\frac{1}{2}} \qquad (S2)$$

and Z_{vib} is the harmonic oscillator vibrational partition function with frequencies ω_i,

$$Z_{vib} = \prod_{i=1} \frac{1}{1 - exp\left(-\frac{\omega_i}{kT}\right)} \qquad (S3)$$

ΔE_n is the zero point vibrational energy ZPVE relative to the smallest ZPVE for each value of n.,

$$\Delta E_n = E_n - E_n^0 \qquad (S4)$$

For the species with n = 1 and 4, there are two formations: internal (int) and external (ext), that is, internal or external location of the minority species H or D, as can be seen in **Scheme S1**.

The probability of finding the species in one of the formations is simply

$$P_n(int) = \frac{Z_n(int)}{Z_n(int) + Z_n(ext)} \qquad (S5)$$

$H_5O_2^+$ (g = 1, σ = 2) 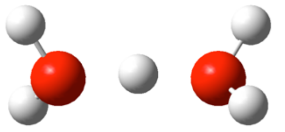	$D_5O_2^+$ (g = 1, σ = 2) 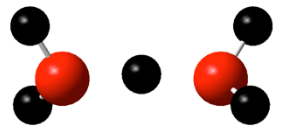
$DH_4O_2^+$ (int) (g = 1, σ = 2) 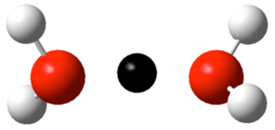	$DH_4O_2^+$ (ext) (g = 4, σ = 1) 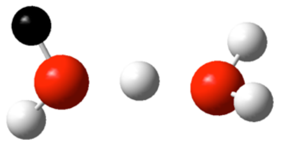
$D_4HO_2^+$ (int) (g = 1, σ = 2) 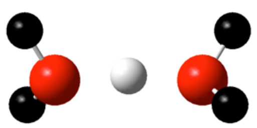	$D_4HO_2^+$ (ext) (g = 4, σ = 1) 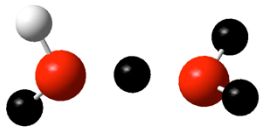

Scheme S1: **Possible formations of $D_nH_{5-n}O_2^+$ with n = 0, 1, 4, 5. Color code: red (O), white (H), black (D)**

S-3 Additional IR and Raman spectra of $H_5O_2^+$ and its deuterium-substituted analogs

This supplementary information presents additional IR and Raman spectra of $H_5O_2^+$ and its deuterium-substituted analogs calculated using the NVE MD simulations. Mixed isotopologues were averaged using the Boltzmann-weighted factors as outlined in the S-2 section.

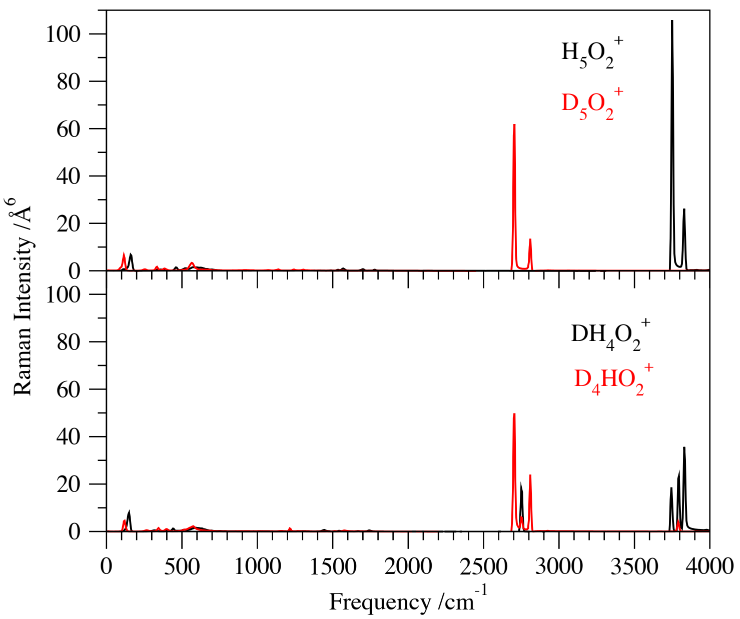

Figure 1S. The total intensities of the Raman spectra for $H_5O_2^+$ and its deuterium isotopologues were calculated at energies corresponding to a temperature of 50 K.

Zoomed-in plots are provided to distinguish between the symmetric in-phase and out-of-phase OH/OD stretches in the high-frequency range Raman spectra of $H_5O_2^+$ (**Figure 2S**) and $D_5O_2^+$ (**Figure 3S**).

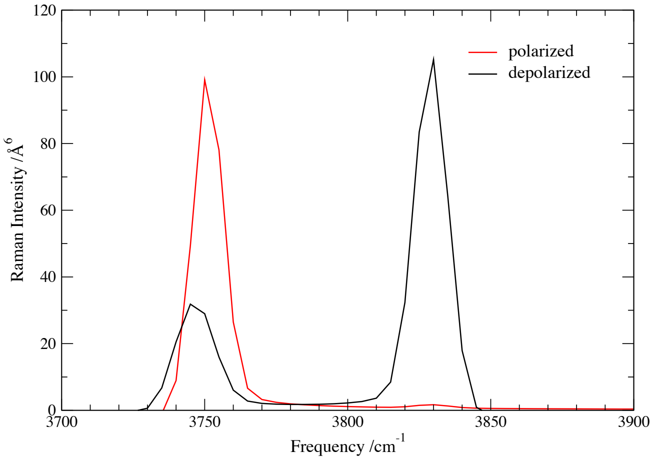

Figure 2S. Zoomed-in plot demonstrating the resolution of two symmetric OH stretches using the polarized (red line) and depolarized (black line) Raman spectra for $H_5O_2^+$ at the energies corresponding to 50 K. The symmetric out-of-phase OH stretch of B symmetry, C_2 point group, appears at 3745 cm$^{-1,}$ while the symmetric in-phase OH stretch of A symmetry appears at 3750 cm^{-1} .

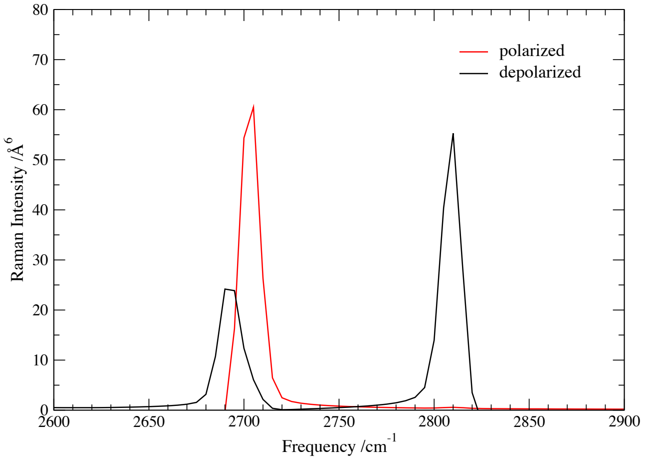

Figure 3S. Zoomed-in plot demonstrating the resolution of two symmetric OD stretches using the polarized (red line) and depolarized (black line) Raman spectra for $D_5O_2^+$ at the energies corresponding to 50 K. The symmetric out-of-phase OD stretch of B symmetry, C_2 point group, appears at 2690 cm$^{-1,}$ while the symmetric in-phase OD stretch of A symmetry appears at 2705 cm^{-1}.

The $DH_4O_2^+$(ext) mixed isotopologue with the D minority species in the external position dominates at all temperatures, which is demonstrated by the IR (**Figure 3,** main text) and Raman spectra (**Figure 4S**), where $DH_4O_2^+$(ext) and $DH_4O_2^+$(aver) are similar.

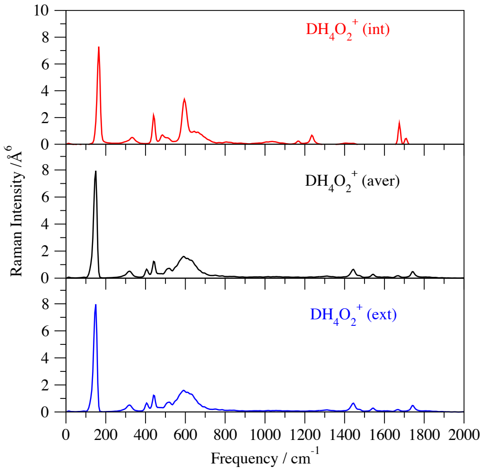

Figure 4S. The total intensities of the Raman spectra for $DH_4O_2^+$. The simulated Raman spectra for the D minority species in the internal (red line) and external (blue line) positions are compared to the averaged $D_4HO_2^+$ spectrum (black line) calculated at energies corresponding to 50 K.

The temperature dependence of the OH/OD stretch in the IR and Raman spectra of $D_4HO_2^+$ (**Figure 5S**) exhibits strong mixing between the (int) and (ext) isotopologues. At low temperature, where $D_4HO_2^+$(int) dominates, there should be only two peaks, one for OD symmetric stretch (overlapping in-phase/out-of-phase components) and one for OD asymmetric stretch (overlapping in-phase/out-of-phase components) (**Table 5S**). At 50 K, there are already three OD stretch peaks and one OH stretch visible due to broken symmetry. Their relative intensity gradually changes as temperature increases, as $D_4HO_2^+$(ext) gains dominance (**Table 6S**).

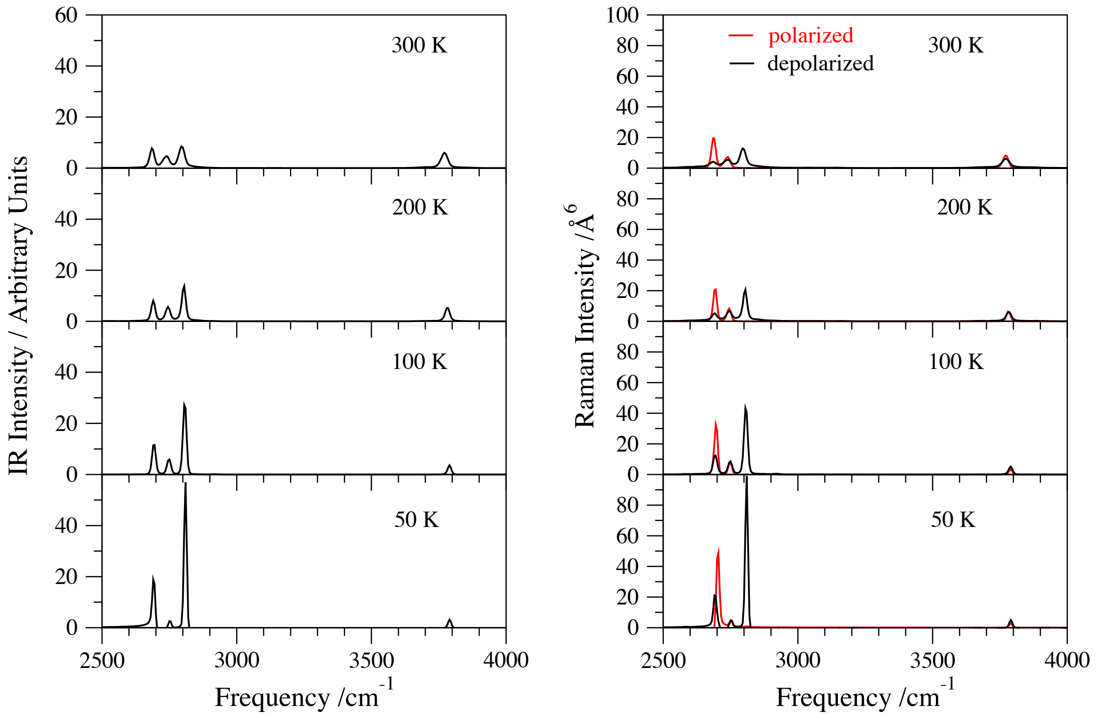

Figure 5S. High-frequency range IR spectra (left panel) and Raman polarized (red line) and depolarized (black line) spectra (right panel) of the $D_4HO_2^+$ deuterium isotopologue calculated at energies corresponding to the temperatures from 50 to 300 K.

References

[1] Huang, X.; Braams, B.J.; Bowman, J. M., Ab initio potential energy and dipole moment surfaces for $H_5O_2^+$. *J. Chem. Phys*. **2005**, *122*, 044308.

[2] Gaussian 16, Revision C.01, Frisch, M. J.; Trucks, G. W.; Schlegel, H. B.; Scuseria, G. E.; Robb, M. A.; Cheeseman, J. R.; Scalmani, G.; Barone, V.; Petersson, G. A.; Nakatsuji, H.; et al. Gaussian, Inc., Wallingford CT, 2016.

[3] MOLPRO, version 2022.2, a Package of *Ab Initio* Programs. Werner, H.-J.; Knowles, P. J.; Knizia, G.; Manby, F. R.; Schütz, M.; Celani, P.; Györffy, W.; Kats, D.; Korona, T.; Lindh, R.; et al. https://www.molpro.net.

[4] McQuarrie, D. A. *Statistical Mechanics*; Harper & Row: New York, 1976.
